# Multirapid Serial Visual Presentation Framework for EEG-Based Target Detection

**DOI:** 10.1155/2017/2049094

**Published:** 2017-07-20

**Authors:** Zhimin Lin, Ying Zeng, Hui Gao, Li Tong, Chi Zhang, Xiaojuan Wang, Qunjian Wu, Bin Yan

**Affiliations:** ^1^China National Digital Switching System Engineering and Technological Research Center, Zhengzhou, China; ^2^Key Laboratory for Neuroinformation of Ministry of Education, School of Life Science and Technology, University of Electronic Science and Technology of China, Chengdu, China

## Abstract

Target image detection based on a rapid serial visual presentation (RSVP) paradigm is a typical brain-computer interface system with various applications, such as image retrieval. In an RSVP paradigm, a P300 component is detected to determine target images. This strategy requires high-precision single-trial P300 detection methods. However, the performance of single-trial detection methods is relatively lower than that of multitrial P300 detection methods. Image retrieval based on multitrial P300 is a new research direction. In this paper, we propose a triple-RSVP paradigm with three images being presented simultaneously and a target image appearing three times. Thus, multitrial P300 classification methods can be used to improve detection accuracy. In this study, these mechanisms were extended and validated, and the characteristics of the multi-RSVP framework were further explored. Two different P300 detection algorithms were also utilized in multi-RSVP to demonstrate that the scheme is universally applicable. Results revealed that the detection accuracy of the multi-RSVP paradigm was higher than that of the standard RSVP paradigm. The results validate the effectiveness of the proposed method, and this method can provide a whole new idea in the field of EEG-based target detection.

## 1. Introduction

A brain-computer interface (BCI) is an advanced human-machine interaction technology that uses a person's electroencephalogram (EEG) and analyzes his/her intentions to interact with the external environment directly. Target image detection based on a rapid serial visual presentation (RSVP) paradigm is a typical BCI application [1, 2].

In the RSVP paradigm, a rapid sequence of images, such as four images per second, is sequentially presented to participants in the same location. As these participants see the target image, they likely induce a special P300 component. The P300 component is a common event-related potential (ERP) component that shows a peak waveform when a small probability event is observed after 300–500 ms [3]. And the P300 component also exhibits significant waveform characteristics in the time domain [4]. Single-trial P300-based systems, such as target image detection based on the RSVP paradigm [1, 5], are commonly used for various BCIs. The P300 component is also detected in the RSVP paradigm to determine the target image of a subject of interest. A P300 detection algorithm is essential because it determines the accuracy and reliability of BCI systems. Farwell and Donchin [6] proposed P300 Speller and used a stepwise linear discriminant analysis (SWLDA) algorithm to detect P300 components. Krusienski et al. [7] compared the performances of various P300 detection algorithms and concluded that SWLDA and Fisher's linear discriminant (FDA) are suitable for the P300 Speller system.

In the RSVP paradigm, the latency and amplitude of P300 components may vary with different experimental parameters [8], such as target probability and stimulus semantics. This variation is a great challenge for single-trial EEG classification in RSVP tasks. To overcome this problem, many scholars proposed effective single-trial detection algorithms. For example, a common spatial pattern is an approach used to search for spatial filters that maximize the variance across two categories [9], such as target and nontarget. Rivet et al. [10, 11] proposed the xDawn algorithm designed to maximize the difference in the signal-to-noise ratio between target and nontarget classes. Bigdely-Shamlo et al. [12] adopted spatial independent component analysis specifically for the single-trial classification of RSVP data to extract a set of spatial weights and obtain maximally independent spatial-temporal sources. Gerson et al. [13–16] proposed the hierarchical discriminant component analysis (HDCA) algorithm to separate single-trial EEG signals into several time windows and to calculate the spatial filter for each time window. Alpert et al. [17] proposed the hierarchical discriminant principal component analysis (HDPCA) algorithm, which introduces principal component analysis for dimensionality reduction. Marathe et al. [18, 19] developed the sliding HDCA (sHDCA) algorithm, which involves standard HDCA evaluation formulated in a typical P300 interval (300–600 ms), and a standard HDCA classifier is slid on single-trial EEG to form a score signal. With this special method of dimension reduction, the imperceptible variation latency of P300 in single-trial EEG data can adapt to the different conditions of subjects. However, the sHDCA algorithm is complex, and its computing speed is relatively slower than that of HDCA. Cecotti et al. [20] developed a spatiotemporal filter that uses the map matrix of a convolutional neural network classifier input layer to a second hidden layer. These algorithms are effective single-trial detection methods, and the target image is assumed to appear only once.

The robustness and stability of multitrial-based P300 component detection are valuable compared to those of single-trial detection [21]. BCI systems based on multitrial detection have been used for extensive applications, such as P300 Speller. However, in target detection application, obtaining images repeatedly is inappropriate for the RSVP paradigm because this method is time-consuming and unconducive to real-time target detection. To solve this problem, Cecotti [22] proposed a dual-RSVP paradigm for target recognition and obtained good results from magnetoencephalography (MEG) data. In dual-RSVP paradigm, two image sequences are simultaneously presented on a screen. One of the image sequences is generated by another image sequence that is delayed for a certain time; hence, the image can appear twice. In this paper, we verified the feasibility of the dual-RSVP paradigm proposed by Cecotti in EEG data and further proposed a triple-RSVP paradigm. In the triple-RSVP paradigm, images can appear thrice in the left, right, and bottom sides of the screen.

On the basis of previously described methods and paradigms, we characterized the components of a multi-RSVP paradigm and revealed their contributions to improve P300 detection accuracy in EEG responses. In this study, the P300 response mechanism in the multi-RSVP framework was revealed and possible problems related to quadruple-RSVP or more conditions were discussed for further improvement. In our experiments, two different P300 detection algorithms were used to demonstrate that the proposed multi-RSVP framework works valuably compared to the traditional RSVP paradigm for EEG-based target detection.

The remaining parts of this paper are organized as follows. First, we present the general rationale for the target detection in the RSVP paradigm. Second, we discuss the specific visual stimulus methods used in this study. Third, we describe the experimental methods. Fourth, we show the classification methods and performance evaluation metrics. Finally, we interpret the results.

## 2. Methods

### 2.1. Visual Stimuli and Procedure

**Figure 1:**
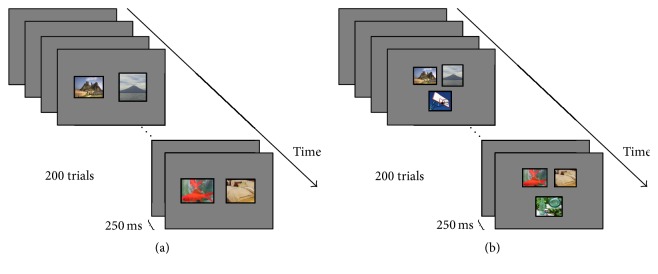
(a) Dual-RSVP: two images were simultaneously presented at the screen left side and right side. (b) Triple-RSVP: three images were simultaneously presented at the screen left, right, and bottom sides. The images were shown in blocks of 200 and flashed at 4 Hz (250 ms).

The participants were seated at 75 cm from a monitor. Images were selected from the ILSVRC15 [23], and the types of images include architecture, birds, artifacts, fruits, aquatic organisms, and natural scenes. The target image category was architecture. These images were presented to the subjects under the dual-RSVP and triple-RSVP paradigms (Figure 1).

**Figure 2:**
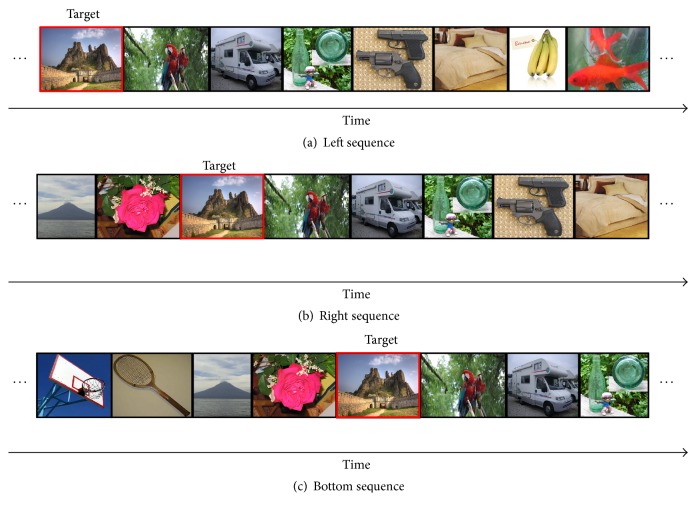
(a) Original image sequence was presented to the left side of the screen. (b) Delayed image sequence was displayed on the right side of screen. (c) Longer delayed image appeared on the bottom side of screen. The dual-RSVP includes (a) and (b). The triple-RSVP includes (a), (b), and (c).

**Figure 3:**
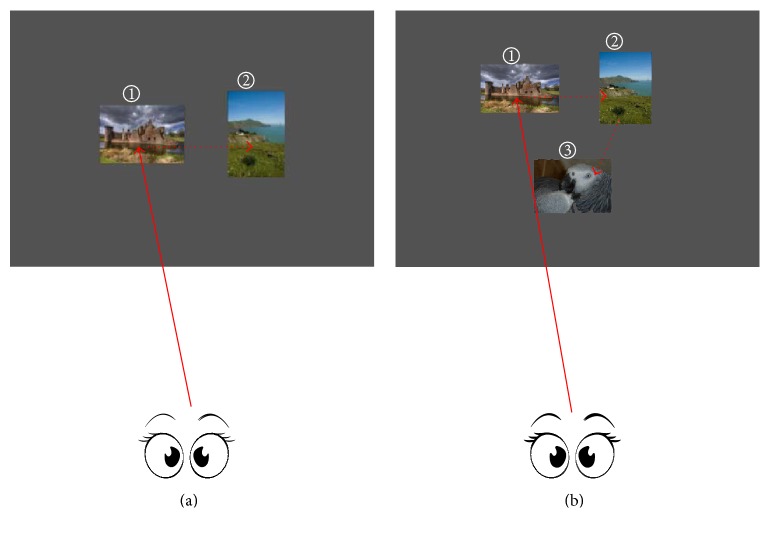
(a) Dual-RSVP: first, the subjects focused on the left image sequence until the target image appeared. The subjects' attention was diverted to the right image sequence until the same target image appeared on this side. Finally, the attention was diverted back to the left side. (b) Similar to (a), however, the difference is that the subjects' attention should be diverted to the bottom side.

Dual-RSVP is a novel experimental paradigm with target detection proposed by Cecotti [22]; the image sequence shown on the left side was presented again on the right side of screen after a certain delay (Figures 2(a) and 2(b)). The subjects gazed at the image stream on the left side until the target image was presented. The subjects then shifted their attention to look at the image stream on right side until the same target was presented. Finally, the subjects focused their attention back to the left side (Figure 3(a)). Cecotti applied this method in the MEG data and achieved good results. In this paper, we propose an improved form of dual-RSVP: triple-RSVP. Similarly, the triple-RSVP simultaneously presents three images (Figure 1(b)). The right images are formed by delaying the left images for a short period of time, and the bottom images are formed by delaying the right images for a period of time (delaying the left images for longer time, Figures 2(a)–2(c)). The subject first looks at the left side, then at the right side, and finally at the bottom side; when the target image is noticed, the subject finally diverts his/her attention back to the left side (Figure 3(b)). In dual-RSVP or triple-RSVP paradigm, the same target image repeatedly appears, and some images are missed in the process of diversion. The probability of the continuous appearance for some target images is small, and the missed images mainly are nontargets. Therefore, the RSVP sequence does not show this condition in our design.

The images were shown in blocks of 200 and flashed at 4 Hz. For these tasks, the RSVP sequence consists of 10 blocks (2000 images, i.e., 200 target images and 1800 nontarget images). Each block consists of 20 target images and 180 nontarget images. We set the left image sequence delay time to 750 ms (three images) and the bottom image sequence delay time to 1500 ms (six images), respectively.

### 2.2. Participants

In this paper, two independent experiments were performed, namely, target detections in dual-RSVP and triple-RSVP paradigms. Seven subjects participated in the dual-RSVP paradigm (two females and five males, mean age 20.6 years, standard deviation 1.3 years). Eight subjects participated in the triple-RSVP paradigm (one female and seven males, mean age 20.2, standard deviation 0.8 years); this is shown in Table 1. All of the subjects were students of Zhengzhou University without previous training in the task. The subjects exhibited normal or corrected-to-normal vision with no neurological problems and were financially compensated for their participation.

### 2.3. EEG Acquisition and Preprocessing

EEG data were acquired by a g.USBamp system (G.Tec company) using 16 electrodes distributed in accordance with the international 10–20 system. In this experiment, the electrooculographic (EOG) will be introduced, because the subjects were asked to transfer the line of sight, when the subjects see the target. In order to ensure the accuracy of the experimental results, we need to remove the eye artifacts before analyzing the data. The EOG activity was recorded by two electrodes positioned above and below the left eye. We collected a group EOC samples before the experiment and implemented the EOC artifact removal by using the method proposed by Zhang et al. [24, 25].

The EEG data were sampled at 2400 Hz using 200 Hz low-pass and 50 Hz notch filters. Prior to scoring the images, we preprocessed the EEG data through the following steps: band-pass filtering (0.5–60 Hz), downsampling to 600 Hz, and baseline correction. Afterwards, the EEG data were divided into epochs of 1000 ms after the stimulus onset.

### 2.4. EEG Analysis

To evaluate the effectiveness of the proposed method, we used the SWLDA and HDCA algorithms to analyze the EEG data. The SWLDA algorithm is a traditional and effective P300 detection algorithm. Farwell and Donchin [6] used the SWLDA algorithm to build the first P300 Speller, and Krusienski et al. [7] reported that SWLDA is the most effective early method of P300 detection. The HDCA algorithm is a new method of P300 detection in the RSVP experiment described by Gerson et al. [13–16]. Many scholars proposed various improved algorithms based on HDCA. The results for SWLDA and HDCA algorithms are a final interest score of each image. We averaged the interest score of the same image in different image sequences (left, right, and bottom sequences). We specified a threshold greater than the threshold value, that is, the target image.

#### 2.4.1. SWLDA Algorithm

The SWLDA algorithm is a feature reduction algorithm that selects suitable features to be included in the discriminant function. The input features are weighted through least square regression to predict the target class labels. First, the most statistically significant initial feature was added to the discriminant function. After each new entry to the discriminant function, a backward stepwise analysis was performed to remove the least significant input features. This process was repeated until no remaining feature satisfies the inclusion/exclusion criteria.

In this paper, the EEG data were divided into epochs. Each epoch consists of 1000 ms EEG data. Epochs corresponding to a certain image were concatenated by each channel to construct a feature vector (14 channels and 60 sample points of each channel; a total of 840 points in a feature vector). We classified the feature vectors by SWLDA and calculated the score for each image.

#### 2.4.2. HDCA Algorithm

The HDCA algorithm was divided into two layers. First, the HDCA algorithm was used to calculate the average data and divide the original EEG data by the time window size. The weight of each channel was then calculated in each time window to maximize the differences between the target and nontarget classes, such as in

(1) $$\begin{array}{c} y_{k}=\left(\;\frac{1}{N}\;\right)\underset{n}{\sum }\underset{i}{\sum }w_{ki}x_{i\left[\left(k-1\right)N+n\right]}, \end{array}$$

where *x*_*i*[(*k* − 1)*N* + *n*]_ represents the *k*th separate time window value from the single-trial data. The variable corresponds to the EEG activity at the data sample point *n* measured by electrode *i*. *w* is a set of spatial weights. Weight vector *w*_*ki*_ is found for the *k*th window and *i* electrode following each image presentation (*T* is the temporal resolution of the time window and in this paper is 0.025, *N* is the sampling time point of the time window, *F*_*S*_ is the sampling rate, *K* is the number of time windows, and *n* = 1,2,…, *N*, *N* = *T* × *F*_*S*_, 0 ≤ *k* ≤ *K*). And *y*_*k*_ is the signal after reduced dimension in *k*th separate time window. In our study, the time window size cannot be determined in advance. Thus, we chose 25 ms as the time window size after numerous experimental repetitions. The weight of each channel in each time window was calculated by Fisher's linear discriminant (FLD).

(2) $$\begin{array}{c} y_{IS}=\underset{k}{\sum }v_{k}y_{k}. \end{array}$$

The results for the separate time windows (*y*_*k*_) were then combined in a weighted *y*_*k*_ average to provide a final interest score (*y*_IS_) for each image. FLD analysis was used to calculate the spatial coefficient *w*_*ki*_, and logistic regression was adopted to calculate for the temporal coefficient *v*_*k*_, such as in (2).

In this paper, the time window size is 25 ms, *k* = 40.

### 2.5. Evaluation of the Algorithm Performance

A tenfold cross-validation was conducted to determine the accuracy of all classification algorithms applied to the EEG data. Performance was evaluated based on the area under the receiver operating characteristic (ROC) curve (AUC) [26].

## 3. Results

### 3.1. Event-Related Responses

**Figure 4:**
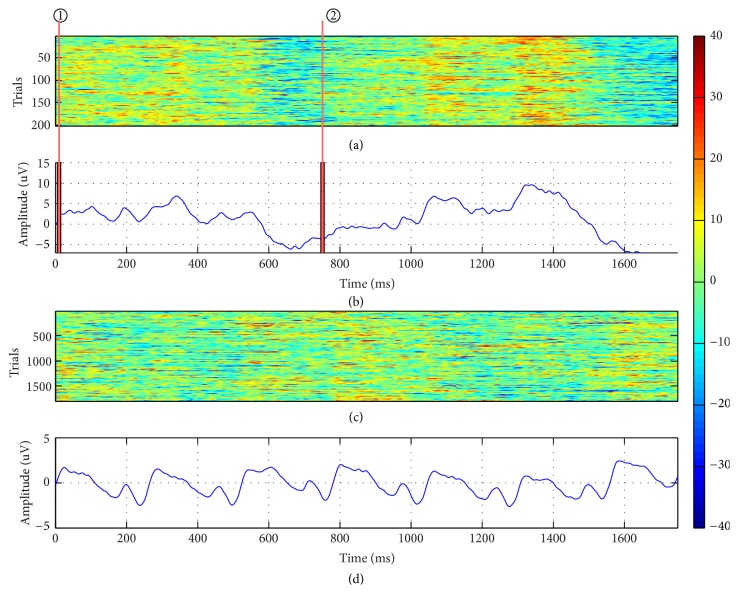
ERP induced by dual-RSVP. (a) All single-trial ERPs to target images at electrode Pz. (b) Grand averages across all trials of the target EEG signals at electrode Pz. (c) All single-trial ERPs to nontarget images at electrode Pz. (d) Grand averages across all trials of the nontarget EEG signals at electrode Pz. The red line ① marked on (a) and (b) is the time point of the first appearance of the target image on the left side, whereas the red line ② is the time point when the target image appeared again on the right side of the screen.

**Figure 5:**
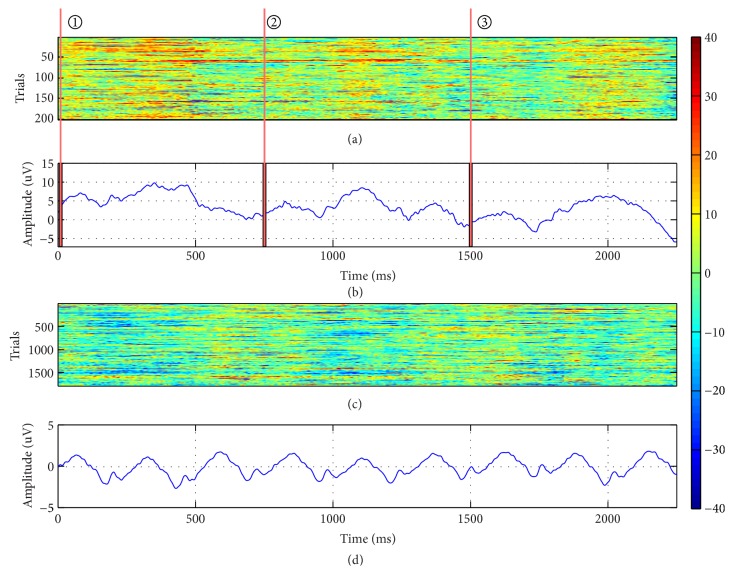
ERP induced by triple-RSVP. (a) All single-trial ERPs to target images at electrode Pz. (b) Grand averages across all trials of the target EEG signals at electrode Pz. (c) All single-trial ERPs to nontarget images at electrode Pz. (d) Grand averages across all trials of the nontarget EEG signals at electrode Pz. The red line ① marked on (a) and (b) is the time point of the first appearance of the target image on the left side, the red line ② is the time point when the target image appeared again on the right side, and the red line ③ is the time point when the target image finally appeared on the bottom side of the screen.

Figure 4 shows the grand mean waveform and all single-trial ERPs induced by dual-RSVP for a specific subject. The *x*-axis is the time of the waveform, and the selected time range is 1750 ms after the first appearance of the target. The target appears twice during this period and thus produces two P300 components. The red line marked on Figures 4(a) and 4(b) is the time point of the target image in the left and right sides of screen, respectively, with the delay of 750 ms (three images). Similarly, Figure 5 shows the grand mean waveform and all single-trial ERPs induced by triple-RSVP in another subject. Three distinct P300 compositions are shown in Figure 5(b), and the *x*-axis time range is 2250 ms after the first appearance of the target. These findings indicate that dual-RSVP and triple-RSVP are valid for P300 and are effective in inducing P300 constituents in the EEG background.

**Figure 6:**
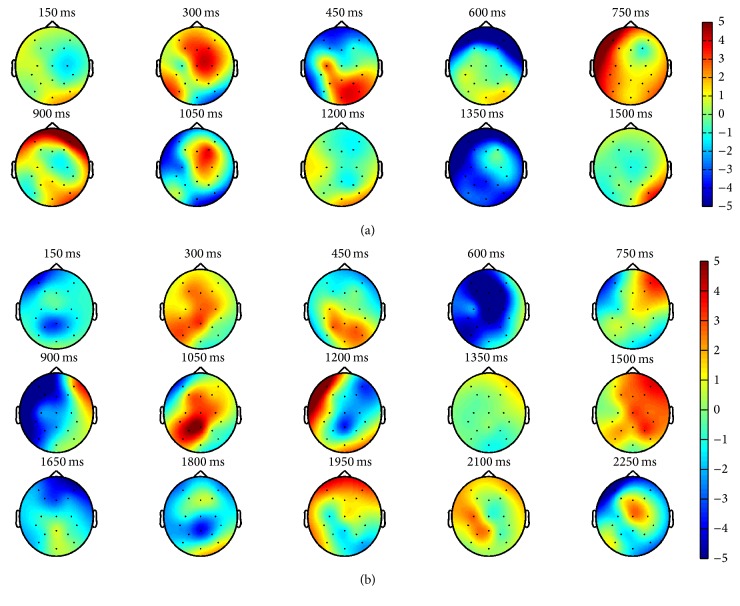
Brain topography induced by dual-RSVP and triple-RSVP. (a) Trend of brain topographic map changes under dual-RSVP paradigm. The target image appeared at 0 ms on the screen left side and at 750 ms on the screen right side, respectively. (b) Trend of brain topographic map changes under triple-RSVP paradigm. The target image appeared at 0 ms on the screen left side, at 750 ms on the screen right side, and at 1500 ms on the screen bottom side, respectively.

Figure 6 further shows the observed brain topography for the target image under the dual-RSVP and triple-RSVP paradigms.  Figure 6(a) shows the dual-RSVP paradigm under the brain topographic map for different time trends. The target image first appeared on the screen left side at 0 ms, followed by the second appearance at 750 ms on the screen right side. In Figure 6(a), a significant P300 activity was observed at 300 and 1050 ms (300 ms after the target occurrence). Similarly, Figure 6(b) shows the triple-RSVP paradigm under the brain topographic map for different time trends. The target image first appeared on the screen left side at 0 ms, followed by the second appearance at 750 ms on the screen right side, and lastly at 1500 ms on the screen bottom side. In Figure 6(b), a P300 component was observed at 300, 1050, and between 1800 and 1950 ms.

### 3.2. Dual-RSVP and Triple-RSVP Performance

We used the AUC value to evaluate the performance of the dual-RSVP and triple-RSVP paradigms. In the dual-RSVP paradigm, we compared the AUC values of the left image sequence EEG score (single-RSVP score) and the combination of the EEG scores from the left and right image sequences (dual-RSVP score) using the HDCA and SWLDA algorithms. Similarly, in the triple-RSVP paradigm, we compared the AUC values of the left image sequence EEG score (single-RSVP score), the combination of the EEG scores from the left and right image sequences (dual-RSVP score), and the combination of the EEG scores from the left, right, and bottom image sequences (triple-RSVP score) using the HDCA and SWLDA algorithms.

Table 2 shows the comparison of AUC values in dual-RSVP paradigm and used the single- and dual-RSVP scores for the seven subjects. Across the subjects, the AUC values of single-RSVP score from the HDCA algorithm are in the range of 0.790–0.929 (mean: 0.882; std: 0.047). The AUC values of the dual-RSVP score from the HDCA algorithm are in the range of 0.803–0.958 (mean: 0.914; std: 0.053). The Wilcoxon signed rank test results are *p* < 0.05. The AUC values of the single-RSVP score from the SWLDA algorithm are in the range of 0.691–0.822 (mean: 0.783; std: 0.062). The AUC values of the dual-RSVP score from the SWLDA algorithm are in the range of 0.729–0.903 (mean: 0.848; std: 0.048). Wilcoxon signed rank test results are *p* < 0.05.

Table 3 shows the comparison of AUC values in triple-RSVP paradigm and the single-, dual-, and triple-RSVP scores for the eight subjects. Across the subjects, the AUC values of the single-RSVP score from the HDCA algorithm are in the range of 0.916–0.952 (mean: 0.926; std: 0.017). The AUC values of the dual-RSVP score from the HDCA algorithm are in the range of 0.935–0.965 (mean: 0.946; std: 0.009). The AUC values of the triple-RSVP score from the HDCA algorithm are in the range of 0.940–0.97 (mean: 0.952; std: 0.008). The Wilcoxon signed rank test results are *p* < 0.05. The AUC values of the single-RSVP score from the SWLDA algorithm are in the range of 0.875–0.917 (mean: 0.908; std: 0.043). The AUC values of the dual-RSVP score from the SWLDA algorithm are in the range of 0.919–0.955 (mean: 0.936; std: 0.037). The AUC values of the triple-RSVP score from the SWLDA algorithm are in the range of 0.936–0.957 (mean: 0.948; std: 0.032). The Wilcoxon signed rank test results are *p* < 0.05.

**Figure 7:**
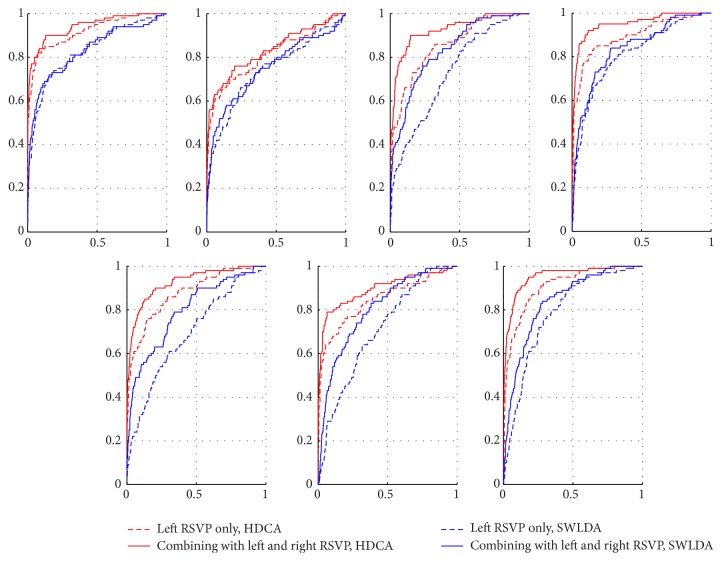
All subjects ROC curve in dual-RSVP paradigm. The red and blue line are the results of HDCA and SWLDA, respectively. The dotted line represents the ROC curve of the left image sequence EEG score. And the solid line represents the ROC curve of combination of the EEG scores from the left image sequence and right image sequences.

**Figure 8:**
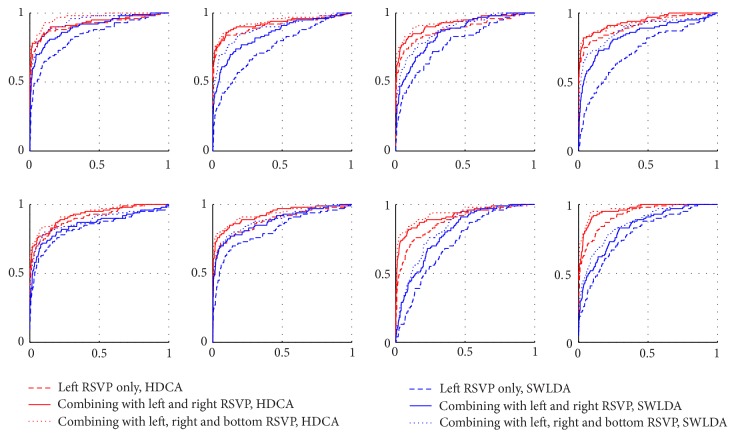
All subjects ROC curve in triple-RSVP paradigm. The red and blue lines are the results of HDCA and SWLDA, respectively. The dotted line represents the ROC curve of the left image sequence EEG score. The solid line represents the ROC curve of the combination of the EEG scores from the left and right image sequences. The small dotted line represents the ROC curve of the combination of the EEG scores from the left, right, and bottom image sequences.

We further plot the ROC curves of all subjects (Figures 7 and 8) in different paradigms (dual-RSVP and triple-RSVP paradigms) and used different P300 detection algorithms (HDCA and SWLDA). We divided the data into two parts, one for training the classifier and one for testing. Figures 7 and 8 show the ROC curves for the test section.  Figure 7 is the ROC curve in dual-RSVP paradigm; the red and blue lines are the results of HDCA and SWLDA, respectively. The results of the combined left and right RSVP sequences are better than those of the left RSVP sequence alone. Analogously, Figure 8 shows the ROC curves for all subjects in triple-RSVP paradigm. The results of the combination of the left, right, and bottom RSVP sequences are better than those of the combination of the left and right RSVP sequences, whereas the combination of the left and right RSVP sequences is better than the left RSVP sequence alone.

## 4. Discussion

The multi-RSVP simultaneously presents multiple images (left side, right side, bottom side, or more of the screen). The right images are formed by delaying the left images for a short period of time, the bottom images are formed by delaying the right images for a period of time, and so on. The subject first looks at the left side, then at the right side, and lastly at the bottom side until the target image appears on each respective side. Finally, the subject focuses again to the left side. Thus, the participant views the target image for multiple times. Experimental results show that this framework effectively improves the accuracy of target recognition.

Obviously, presenting the target image for multiple times effectively improves the accuracy of P300 recognition. In the dual-RSVP paradigm or triple-RSVP paradigm, once the subject sees the target image, he/she naturally acknowledges that the target image will appear again in another sequence after an approximate time. In the cognition of the subject, the probability of the reappearance of the target image increases and thus reduces the induced P300 attribute. As shown in Figure 5(b), in the triple-RSVP paradigm, the peak of the third P300 component is smaller than that of the first and second, and the third P300 latency is longer. This finding indicates that detection of the third P300 component is more difficult than that of the first or second P300 component. Figure 8 and Table 3 show that the increased performance of the triple-RSVP compared with the dual-RSVP is lower than that of the dual-RSVP compared with the single-RSVP. This finding reveals that in the multi-RSVP framework, the ability of quadruple-RSVP (or higher) to increase the accuracy is limited, and the complexity of the operation should also be considered. One possible strategy to improve the multi-RSVP framework is to randomize the reappearance time interval of the target image in another image sequence. Hence, the subject cannot confirm the specific time when the target image reappears. This theory will be studied in a future research.

**Figure 9:**
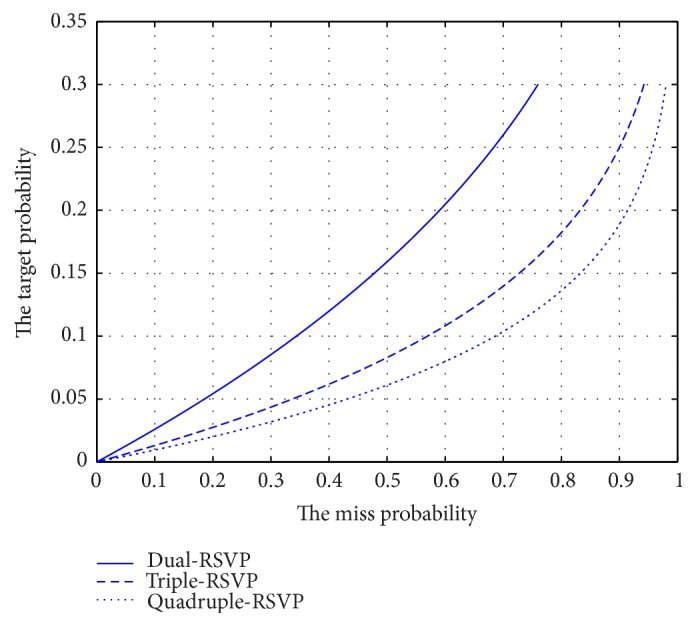
Target appearing probability and target miss probability in dual-, triple-, and quadruple-RSVP paradigms.

In practical applications, we need to consider the conditions where multi-RSVP (dual-, triple-, or more) is applicable. In the experiment, we assumed that the adjacent target image does not appear; however, this phenomenon can occur in practice. In this paper, the probability of appearance of target image is 0.1. In the dual-RSVP paradigm, the subjects observe the same target twice at 1250 ms to 1500 ms, during which the subjects ignore the original image sequence (5-6 images) after the target appears, during which the subjects ignore 5-6 images in the image sequence of the screen left side. Similarly, in the triple-RSVP paradigm, the subject misses 8-9 images. These missed images probably contain the target image. In the quadruple- (or more) RSVP paradigm, the probability of missing the target image is high. Therefore, the probability of target occurrence is an important factor in selecting the appropriate multi-RSVP (dual-, triple-, or more) paradigm.  Figure 9 shows the relationship curve of the probabilities when the target appears and the target is missed. The target miss probability is calculated by

(3) $$\begin{array}{c} P_{miss}=1-{\left(\;1-P_{target}\;\right)}^{N}, \end{array}$$

where *P*_miss_ represents the target miss probability in the process of the subject transfer sight. *N* is the number of ignored images, and *P*_target_ is the target probability.

Thus, the multi-RSVP paradigm is valid only when the probability of target occurrence is low. This finding impedes the application of multi-RSVP paradigm. In practical applications, some scenes can satisfy this condition. For example, the probability of a particular target image (such as the threat image) is extremely low in the Cognitive Technology Threat Warning System [27, 28]. However, serious consequences likely occur when the threat of the target is undetected. Under these conditions, the multi-RSVP paradigm is an effective method. The target image then appears many times and thus ensures the high-precision identification of the target.

## 5. Conclusion

In this study, we verified the feasibility of the dual-RSVP paradigm [22] proposed by Cecotti in EEG data and further established a triple-RSVP paradigm. Multi-RSVP framework is effective for target detection. The multi-RSVP paradigm achieves higher recognition accuracy than the standard RSVP paradigm.

## Figures and Tables

**Table 1:** The human subjects information.

Experiment	Number of participants	Average age	Standard deviation
Dual-RSVP	7 (2 female and 5 male)	20.6	1.3
Triple-RSVP	8 (1 female and 7 male)	20.2	0.8

**Table 2:** Values of the AUC of all subjects under the dual-RSVP paradigm.

	Subjects	1	2	3	4	5	6	7	Mean	SD
Left RSVP only	HDCA	0.9294	0.7901	0.9019	0.8934	0.8813	0.8577	0.9205	0.8820	0.0471
	SWLD	0.8226	0.6917	0.7935	0.8021	0.8074	0.7615	0.8072	0.7837	0.0448

Combining with left and right RSVP	HDCA	**0.9486**	**0.8026**	**0.9409**	**0.9358**	**0.9211**	**0.8964**	**0.9583**	**0.9148**	0.0534
	SWLD	**0.8952**	**0.7293**	**0.8656**	**0.8725**	**0.8461**	**0.8268**	**0.9033**	**0.8484**	0.0588

**Table 3:** Values of the AUC of all subjects under the triple-RSVP paradigm.

	Subjects	1	2	3	4	5	6	7	8	Mean	SD
Left RSVP only	HDCA	0.9337	0.9191	0.9169	0.9306	0.9213	0.9194	0.9186	0.9512	0.9263	0.0118
	SWLD	0.9152	0.9176	0.9121	0.9107	0.9097	0.9140	0.8759	0.9100	0.9082	0.0133

Combining with left and right RSVP	HDCA	0.9477	0.9434	0.9461	0.9448	0.9351	0.9381	0.9466	0.9656	0.9459	0.0091
	SWLD	0.9381	0.9469	0.9460	0.9552	0.9257	0.9228	0.9199	0.9361	0.9363	0.0127

Combining with left, right, and bottom RSVP	HDCA	**0.9572**	**0.9514**	**0.9520**	**0.9482**	**0.9465**	**0.9401**	**0.9520**	**0.9701**	**0.9522**	0.0088
	SWLD	**0.9580**	**0.9542**	**0.9518**	**0.9538**	**0.9448**	**0.9429**	**0.9364**	**0.9458**	**0.9485**	0.0072
